# Overexpression of chemokine receptor lymphotactin receptor 1 has prognostic value in clear cell renal cell carcinoma

**DOI:** 10.1002/mgg3.1551

**Published:** 2020-12-30

**Authors:** Xian Chang, Ya Cao, Wan‐Lei Fu, Xue‐Feng Tang, Ya‐Li Wang, Yang‐Fan Lv, Qiao‐Nan Guo

**Affiliations:** ^1^ Department of Orthopedics Xinqiao Hospital Third Military Medical University (Army Medical University Chongqing P. R. China; ^2^ Department of Pathology Xinqiao Hospital Third Military Medical University (Army Medical University Chongqing P. R. China

**Keywords:** ccRCC, prognosis, STAT, TCGA, XCR1

## Abstract

**Background:**

Clear cell renal cell carcinoma (ccRCC) is an aggressive subtype of renal cell carcinoma. X‐C motif chemokine receptor 1 (XCR1) exerts important roles in tumor progression; however, its role in ccRCC is unclear.

**Methods:**

We utilized publicly available data from The Cancer Genome Atlas (TCGA) to assess the role of *XCR1* in ccRCC and validated the results in 36 samples from patients with ccRCC who underwent curative resection in Xinqiao Hospital Chongqing. XCR1 overexpression was identified in ccRCC, which was confirmed by qRT‐PCR assay and immunohistochemical staining of ccRCC samples.

**Results:**

For the TCGA and clinical data, Kaplan–Meier survival analysis revealed that higher XCR1 expression in ccRCC was related to longer overall survival. Cox regression analysis suggested that XCR1 is an independent risk factor for ccRCC. GSEA analysis suggested that *XCR1* is associated with the JAK/STAT signaling pathway. *XCR1* knockdown by small interfering RNA (siRNA) significantly increased ccRCC cell proliferation and migration, and decreased cell apoptosis.

**Conclusion:**

We found higher XCR1 expression in ccRCC compared with that in normal tissues is related to longer overall survival in patients with ccRCC. XCR1 knockdown significantly increased RCC cells proliferation and migration, and decreased apoptosis. XCR1 might be used as a prognostic biomarker in ccRCC in the future.

## Graphical abstract

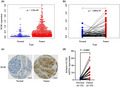

## 1 INTRODUCTION

Renal cell carcinoma (RCC) is the one of the most common malignant tumors of the urinary system, which accounts for ~2% to 3% of all adult malignant tumors (Baggiolini et al., 1997). There were 403262 (2.2%) new patients and 175098 (1.8%) deaths caused by renal cancer around the world in 2018 (Balkwill, 2004). Among types of RCC, ccRCC is the most common subtype, representing 60%–85% of cases according to the World Health Organization (WHO; Blaschke et al., 2003). Progress in medical diagnosis and improvements in health awareness has led to earlier detection and treatments in some patients with RCC, resulting in improved prognosis for patients with ccRCC. However, about one‐quarter patients are diagnosed with progressive stage disease and one‐third of patients experience recurrence after surgery. Once metastasis occurs, the prognosis is extremely poor, with 5‐year survival ranging from 0% to 20%. Despite this low survival rate, the detailed mechanisms underlying ccRCC progression are still unknown; however, identifying effective molecular targets involved in ccRCC carcinogenesis and metastasis to improve the outcomes for patients with ccRCC remains a challenge.

The chemokine super family plays an important role in tumor metastasis. Chemokines interact with chemokine receptors, affecting the whole process of tumor development, including tumor cell growth, angiogenesis, angiostasis, metastasis, and local invasion (Brinckmann et al., 2002; Chen et al., 2019). The lymphotactin receptor X‐C motif chemokine receptor 1 (XCR1) is an important member of the chemokine receptor family. Emerging evidence indicate that the XCR1 binds with its ligand X‐C Motif Chemokine Ligand 1 (XCL1), which is closely related to an organism's immune function (Dorner et al., 2009; Feist et al., 2018). Meanwhile, the XCL1/XCR1 axis also contributes to the progression of various diseases, including rheumatoid arthritis (Gantsev et al., 2013), breast cancer, and non‐small cell lung cancer. XCR1 is overexpressed in ovarian carcinoma, oral cancer, and breast cancer (Hsieh et al., 2017; Jemal et al., 2010; Khurram et al., 2010). XCR1 overexpression could inhibit liver cancer cells’ proliferation, and promote tumor migration (Kim et al., 2012). XCR1 is overexpressed in non‐small cell lung cancer and is associated with its bone metastasis (Lei et al., 2011). Research by He's research group indicated the interleukin (IL) 10, together with XCR1 might be a key regulator for the tumor microenvironment of ccRCC. Besides, no study has as yet comprehensively explored XCR1’s expression and its role in tumor development, especially its prognostic value in ccRCC.

The present study aimed to assess the prognostic value of XCR1 in ccRCC, according to data from The Cancer Genome Atlas (TCGA). Gene set enrichment analysis (GSEA) was used to further explore the role of XCR1 in ccRCC pathogenesis. Moreover, experiments by human histological specimens and ccRCC cell lines were used to validate the results.

## 2 MATERIALS AND METHODS

### 2.1 Ethical compliance

The Ethics Committee of Xinqiao hospital (the Second Affiliated Hospital of Army Military Medical University) approved the study protocol. Each patient provided written informed consent, and this study complied with the Declaration of Helsinki.

### 2.2 RNA‐sequencing, patient data, and bioinformatic analysis

The patients’ clinical parameters, including survival follow‐up information, age, sex, and clinically relevant tumor classification for 539 ccRCC samples and 72 normal samples from the TCGA LIHC cohort were downloaded from the Genomic Data Commons (GDC) data portal and used to analyze the mRNA expression levels. Fifty paired tumor/normal samples were used to analyze differential mRNA expression. Subsequently, the RNA sequencing (RNA‐Seq) gene expression level 3 HTSeq‐Counts data of 539 patients with ccRCC and the clinical data were retained and further analyzed. Genes in this study are XCR1 (NM_001024644.2).

### 2.3 Tissue samples

Thirty‐six pairs of fresh tissue samples comprising ccRCC and adjacent non‐cancerous tissues were obtained from patients who underwent radical or partial nephrectomy in Xinqiao Hospital Army Medical University and stored in liquid nitrogen and.

### 2.4 Cell culture

The human RCC cell line 786‐O was purchased from the ATCC (Manassas, VA, USA) and was cultured in Roswell Park Memorial Institute (RPMI)‐1640 medium supplemented with 10% fetal bovine serum (FBS).

### 2.5 RNA extraction, reverse transcription, and quantitative real‐time PCR

Total RNA was extracted from frozen tissues and cultured cells using the Trizol reagent (Invitrogen) according to the manufacturer's instructions. mRNA reverse transcription and quantitative real‐time PCR were executed using an SYBR Green Kit (Invitrogen) with an ABI PRISM 7500 system (Applied Biosystems). The *GAPDH* gene (glyceraldehyde‐3‐phosphate dehydrogenase) was used as an internal control and the other primers applied to quantitative real‐time reverse transcription PCR (qRT‐PCR) are shown in Table 1.

**Table 1:** Primer pairs used for real‐time PCR.

Gene	Forward primer sequence (5′ to 3′)	Reverse primer sequence (5′ to 3′)
*GAPDH*	CGCTGAGTACGTCGTGGAGTC	GCTGATGATCTTGAGGCTGTTGTC
*XCR1*	TACCTCACCTCCGTCTACCA	GATGAGCAGGGCGTATTCTA

XCR1, X‐C motif chemokine receptor 1.

### 2.6 Immunohistochemistry

Immunohistochemistry (IHC) was implemented in renal tumor tissue sections using a previously described method (Lv et al., 2016). Briefly, after deparaffinization, hydration, and antigen retrieval, the sections were incubated overnight with a primary antibody recognizing XCR1 (dilution 1:80; cat. no. 2019; Cell Signaling Technology, Inc.) at 4°C. Subsequently, secondary antibodies were applied after washing, and the nuclei were counterstained with hematoxylin. The sections were observed and photographed under an Olympus BX50 light microscope (Olympus). Specimens were independently assessed by two pathologists who were blinded to both clinical and pathological data. For the semi‐quantitative assessment of XCR1 expression, the percentage of positive ccRCC cells was calculated in more than five randomly selected fields of view with higher magnification objectives and included over 100 cells. The final score is a product of the positive cell ratio score (0, 0% to 10% positive; 1, 10%–50% positive; 2, 50%–80% positive; 3, >80% positive) and relative expression score (1, yellow staining; 2, brown staining; 3, dark brown staining). Final scores ≥3 were considered positive.

### 2.7 Gene set enrichment analysis

GSEA is a calculation method that involves deleting a gene from a gene set that is expressed in less than 80% of samples, to provide an *a priori* defined set of genes that show statistically significant, concordant differences between two biological states (Lv et al., 2015). In this study, GSEA generated an ordered list of all genes correlated with XCR1 differential expression, generating groups with markedly high and low expression of *XCR1*. Gene set permutations were carried out 1000 times for each analysis. The phenotype label was formulated using the expression levels of *XCR1*. The normalized enrichment score (NES) and nominal *P* value were used to rank the pathways enriched for each phenotype.

### 2.8 Construction of a siRNA vector targeting XCR1 (siXCR1)

The siXCR1 sequences were 5′‐CCCUCACCAACAUCUUCAUTT‐3′ and 5′‐AUGAAGAUGUUGGUGAGGGTT‐3′. The non‐target control siRNA sequence was 5′‐TTCTCCGAACGTGTCACGT‐3′.

### 2.9 Western blotting, CCK‐8 assays, trans‐well migration, and flow cytometry assays

Western blotting, cell counting kit 8 (CCK‐8), Transwell migration, and flow cytometry assays were performed as previously described (Patel et al., 2006). The primary antibodies used in this study were as follows: GAPDH (dilution 1:1000; Cell Signaling Technology, Inc.), XCR1 (dilution 1:600; Cell Signaling Technology, Inc.), JAK1 (dilution 1:600; cat. no. 2019; Cell Signaling Technology, Inc.), phospho‐JAK1 at Tyr1034/1035 (dilution 1:500; Cell Signaling Technology, Inc., JAK2 (dilution 1:1000; Cell Signaling Technology, Inc.), phospho‐JAK2 at Tyr1007 (dilution 1:1000; Cell Signaling Technology, Inc.), JAK3 (dilution 1:500; Cell Signaling Technology, Inc.), phospho‐JAK3 at Tyr980 (dilution 1:500; Cell Signaling Technology, Inc.), STAT 1–4 (dilution 1:500; dilution 1:500; Stat Antibody Sampler Kit, Cell Signaling Technology, Inc.), phospho‐STAT1 at Tyr701, phospho‐STAT2 at Tyr690, and phospho‐STAT4 at Tyr693 (dilution 1:500; Cell Signaling Technology, Inc.).

### 2.10 Statistical analysis

The R software (v.3.4.3; https://www.r‐project.org/) was used carried out the bioinformatic analysis. Kaplan–Meier analysis was used to analyze the clinicopathological features associated with overall survival in patients with ccRCC. Both univariate and multivariate Cox analyses were adopted to investigate the prognostic value of *XCR1* and the indicated clinicopathological characteristics in ccRCC. Cox regression was adopted to assess the hazard ratio (HR) and 95% confidence interval (CI). The cut‐off value of *XCR1* expression was determined by its median value. Statistical analyses in the cytological experiments were performed using the SPSS 22.0 software package (version 22.0; SPSS Inc.). At least three independent experiments were applied and all data were presented as mean ± SD. Student's *t*‐test was used for comparisons between two groups and *p* < 0.05 was considered statistically significant. For association analysis between IHC samples and clinical‐pathological parameters analysis, the Pearson χ^2^ test was used.

## 3 RESULTS

### 3.1 Patient characteristics

The characteristics of the patients from the TCGA dataset and the Xinqiao Hospital Army Medical University are shown in Table 2, comprised of age, diagnosis, sex, tumor grade, TNM stage, follow‐up month, and survival condition of the patients.

**Table 2:** Clinicopathological features of patients included in this study.

	TCGA database	Clinical Samples
Total	539	36
Age	26–90 (61)	24–78 (56)
**Gender**		
Male	348	23
Female	191	13
**Grade**		
G1	14	3
G2	230	13
G3	207	15
G4	78	5
NA	10	0
**TNM staging**		
Ⅰ	269	11
Ⅱ	57	15
Ⅲ	125	8
Ⅳ	83	2
NA	5	0
**Follow‐up, months**	0–121	20–83
**Survival condition**		
Dead	172	8
Alive	367	28

### 3.2 XCR1 is overexpressed in ccRCC

**FIGURE 1:**
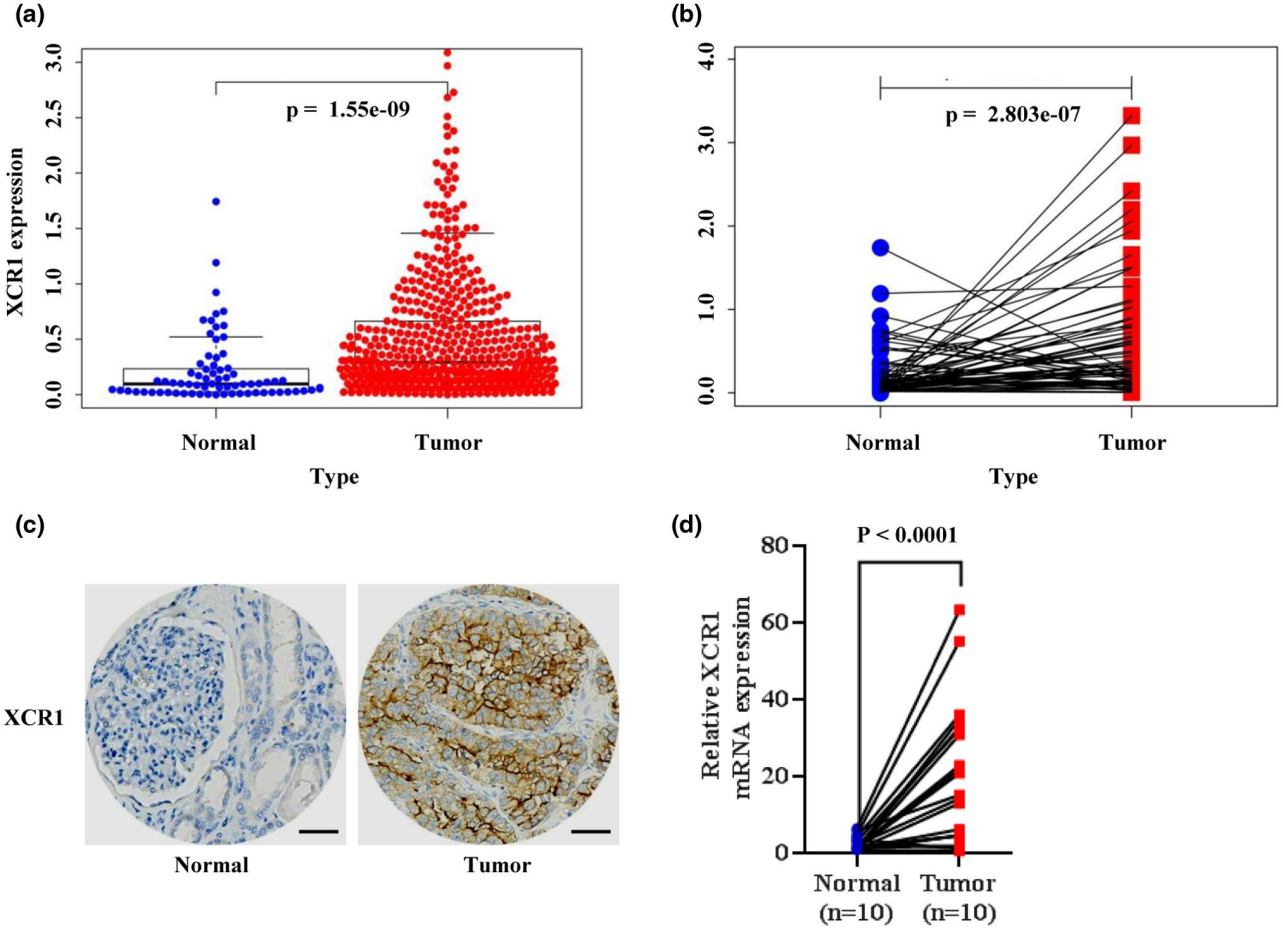
*XCR1* is overexpressed in ccRCC. (a) ccRCC (n = 539) samples compared with normal kidney (n = 72) samples based on the TCGA data (unpaired *t*‐test, *p* = 1.55e‐09) showing that the expression levels of *XCR1* were upregulated in ccRCC. (b) ccRCC samples compared with paired adjacent normal samples according to the TCGA data, suggesting that the expression levels of *XCR1* were upregulated in ccRCC (paired *t*‐test, *p* = 2.803e‐07). (c) Representative images of IHC of XCR1 in ccRCC clinical samples and paired adjacent normal kidney tissues. Scale bars: 50 μm. (d) In tumor tissue samples, XCR1 was overexpressed compared with that in paired adjacent normal tissue samples (paired *t*‐test, *p* < 0.0001).

*XCR1* expression in ccRCC and normal kidney tissues in patients from the TCGA dataset was evaluated. As shown in Figure 1, *XCR1* was significantly upregulated in ccRCC compared with normal kidney samples (*p* = 1.55e‐09, Figure 1a). Moreover, *XCR1* was highly expressed in ccRCC tissues compared with that in paired adjacent normal tissues (*p* = 2.083e‐7, Figure 1b). The IHC staining results of the 36 clinical samples also showed that the expression of XCR1 was significantly higher in tumor tissues compared with that in normal tissue (Figure 1c). Next, qRT‐PCR was applied to detect the expression of *XCR1* in ccRCC samples and paired adjacent normal kidney tissues, and *GAPDH* was used as the internal control. The result further validated that *XCR1* was highly expressed in ccRCC (*p* < 0.001, Figure 1d).

### 3.3 Correlation between *XCR1* expression and ccRCC malignancy

**FIGURE 2:**
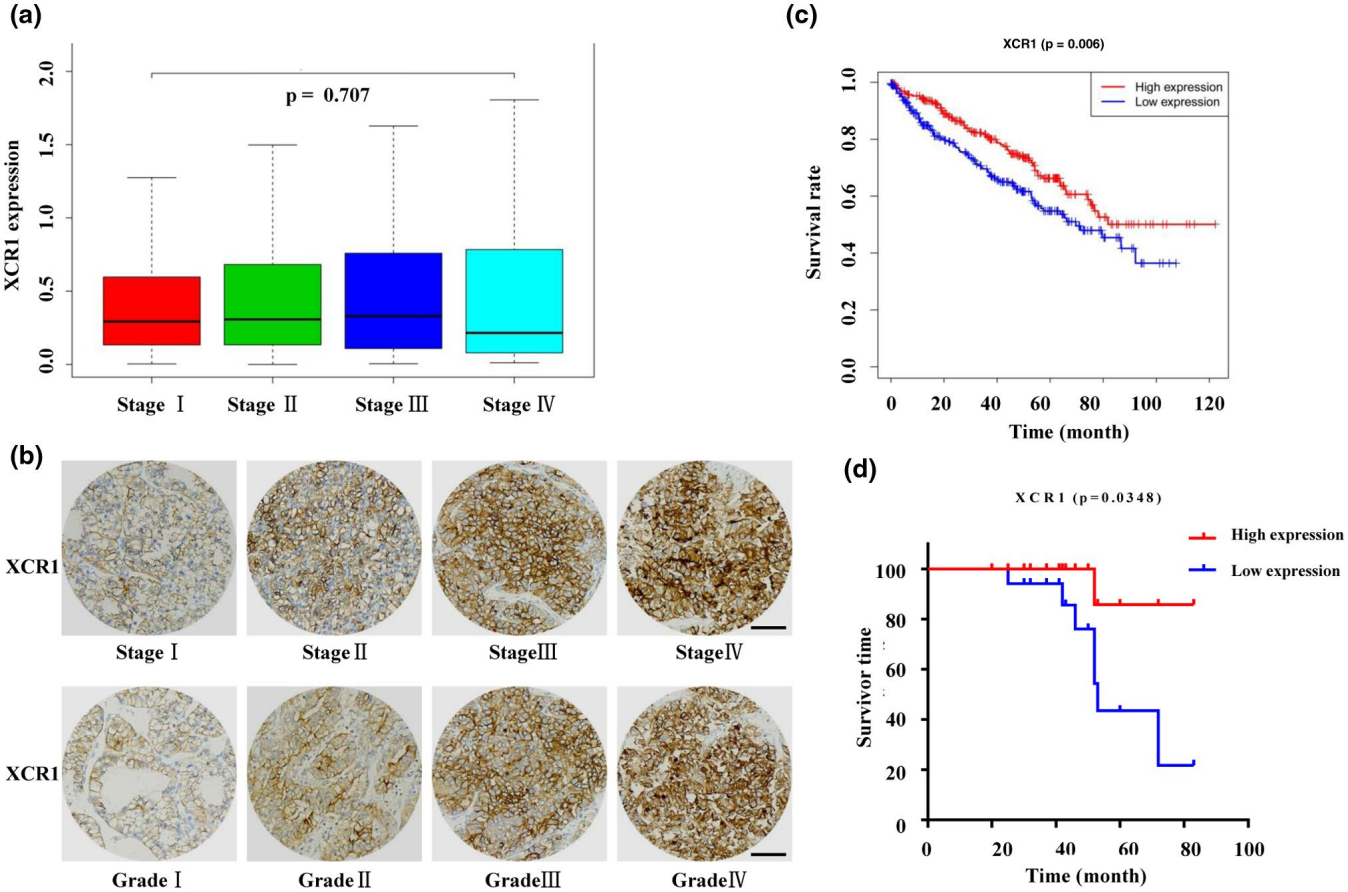
The XCR1 expression profile is associated with survival in ccRCC. (a) Box plot showing the correlation between XCR1 expression and ccRCC stages (*p* = 0.707). (b) Representative images of XCR1 expression examined by IHC in different stages and grades of ccRCC specimens. Scale bars: 50 μm. (c) The data from the TCGA database were used to analyze *XCR1* expression levels in ccRCC patients using Kaplan–Meier survival analysis. There was a significantly better cancer‐specific survival rate in patients with high *XCR1* expression compared with that in patients with low *XCR1* expression (*p* = 0.006). (d) The XCR1 expression levels in ccRCC clinical samples analyzed using Kaplan–Meier survival analysis. Significantly longer overall survival was observed in patients with high XCR1 expression (*p* = 0.0348).

Based on data from the TCGA, we found that from stage Ⅰ to Ⅲ, with tumor progression, the expression of *XCR1* slightly increased; however, there was no statistical difference in the overall trend (Figure 2a). We then assessed XCR1 expression in 36 clinical samples with ccRCC using IHC staining. The IHC of XCR1 showed to be membrane positive and gradually increased from stage Ⅰ to Ⅳ; moreover, its expression in the tumor of grade Ⅰ is very weak or invisible, while that in grade Ⅱ to Ⅳ is much stronger (Figure 2b). Further statistical results showed XCR1 expression was significantly associated with the TNM stage of ccRCC (*p* < 0.05, Pearson χ^2^ test; Table 3), whereas the results related to age, gender, and tumor grade are negative (Table 3). Kaplan–Meier survival analysis showed XCR1 expression levels were closely related to overall survival in patients with ccRCC patients, with higher XCR1 levels being associated with longer overall survival (*p* = 0.006 Figure 2c). Moreover, Kaplan–Meier survival analysis based on the data from the 36 clinical samples from Xinqiao hospital also validated the prognostic value of XCR1 in ccRCC: A higher level of XCR1 correlated significantly with better overall survival (*p* = 0.0348, Figure 2d).

**Table 3:** Association between XCR1 expression and the clinic‐pathological characteristics of clinical samples from ccRCC patients.

Characteristics	Positive	Negative	P‐value
**Age**			
<45	7	4	0.984
≥45	16	9	
			
**Gender**	14	9	
Male	9	4	0.621
female			
**Grade**	2	1	
Ⅰ	5	8	0.125
Ⅱ	12	3	
Ⅲ	4	1	
Ⅳ			
**TNM staging**	8	3	
Ⅰ	3	12	0.019
Ⅱ	1	7	
Ⅲ	1	1	
Ⅳ			

**FIGURE 3:**
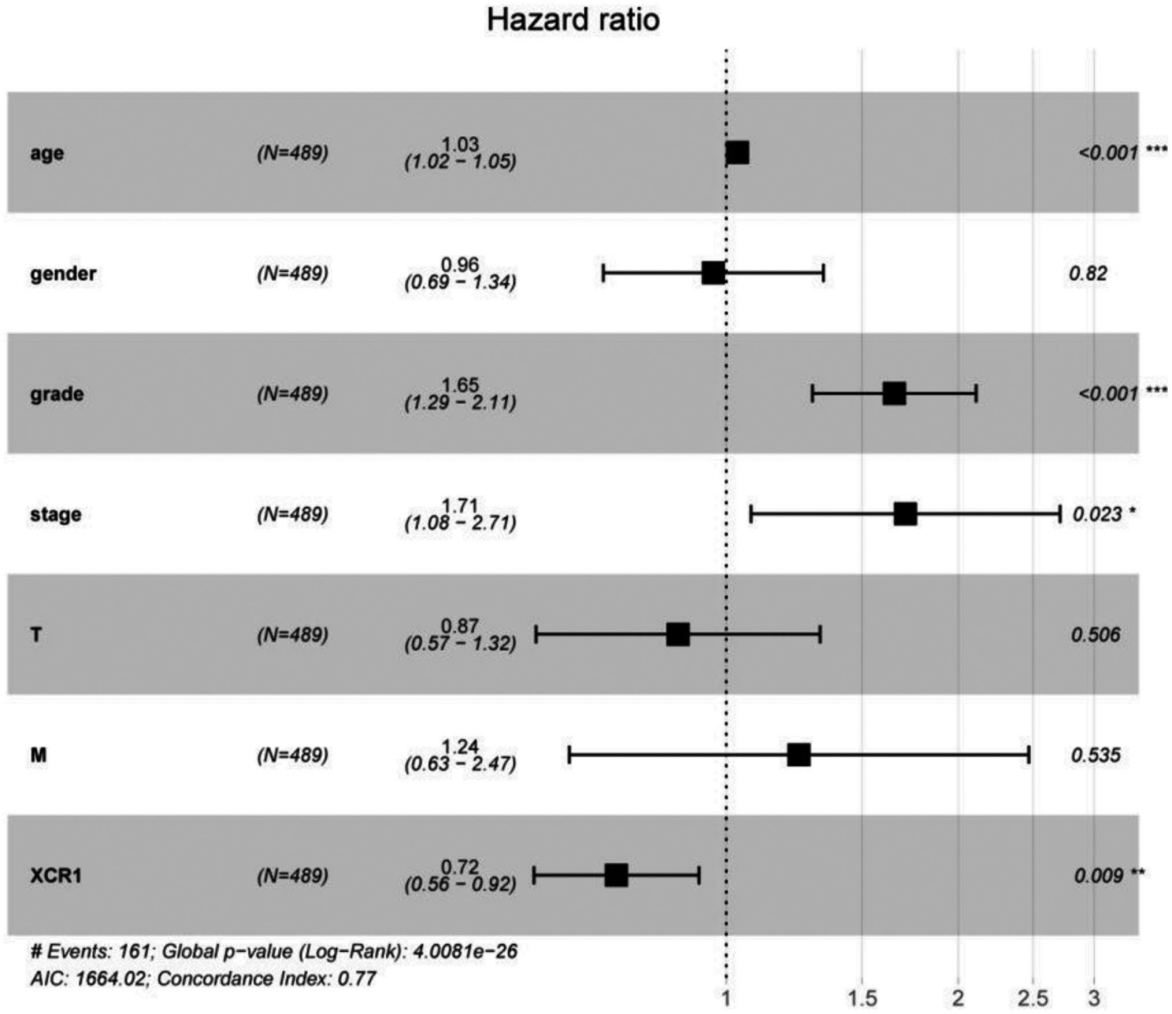
Forest plot summary of the analyses of overall survival (OS). Multivariate analyses of age, sex, grade, stage, and XCR1 expression group for OS in patients with ccRCC. The black squares on the transverse lines represent the hazard ratio (HR), and the black transverse lines represent the 95% confidence interval (CI).

Subsequently, univariate and multivariate Cox regression analyses were performed to further understand the prognostic significance of XCR1 in ccRCC. Univariate analysis indicated that age (*p* = 2.29E‐06), tumor grade (*p* = 1.94E‐14), tumor stage (*p* = 4.67E‐20), T (*p* = 1.50E‐14), and M (*p* = 7.45E‐19) of the tumor‐node‐metastasis (TNM) system all correlated with OS. When all these factors were included in the multivariate analysis, age (*p* = 1.44E‐05), tumor grade (*p* = 5.60E‐05), and tumor stage (*p* = 0.022938) continued to be significantly associated with OS. Moreover, XCR1 appeared to be a protective prognostic marker for OS in patients with ccRCC patients (HR=0.721; *p* = 0.009, Table 4, Figure 3).

**Table 4:** Cox regression analysis of XCR1 mRNA levels and patient overall survival with ccRCC.

Variable	Univariate analysis				Multivariate analysis			
	HR	HR.95L	HR.95H	**P value**	HR	HR.95L	HR.95H	**P value**
age	1.032826	1.019083	1.046754	**2.29E−06**	1.034216	1.018615	1.050057	**1.44E−05**
Sex	0.931081	0.675354	1.283641	**0.662937**	0.962538	0.692753	1.337389	**0.820013**
Grade	2.293061	1.854092	2.835959	**1.94E−14**	1.652044	1.294088	2.109014	**5.60E−05**
Stage	1.888786	1.648774	2.163737	**4.67E−20**	1.707664	1.076801	2.708128	**0.022938**
T	1.94139	1.639292	2.29916	**1.50E−14**	0.86604	0.566668	1.32357	**0.506312**
M	4.283544	3.105734	5.908024	**7.45E−19**	1.242764	0.625942	2.467424	**0.534532**
XCR1	0.815312	0.638049	1.041823	**0.102594**	0.720596	0.56334	0.921749	**0.00909**

HR, hazard ratio

### 3.4 GSEA identified XCR1‐related signaling pathways

**FIGURE 4:**
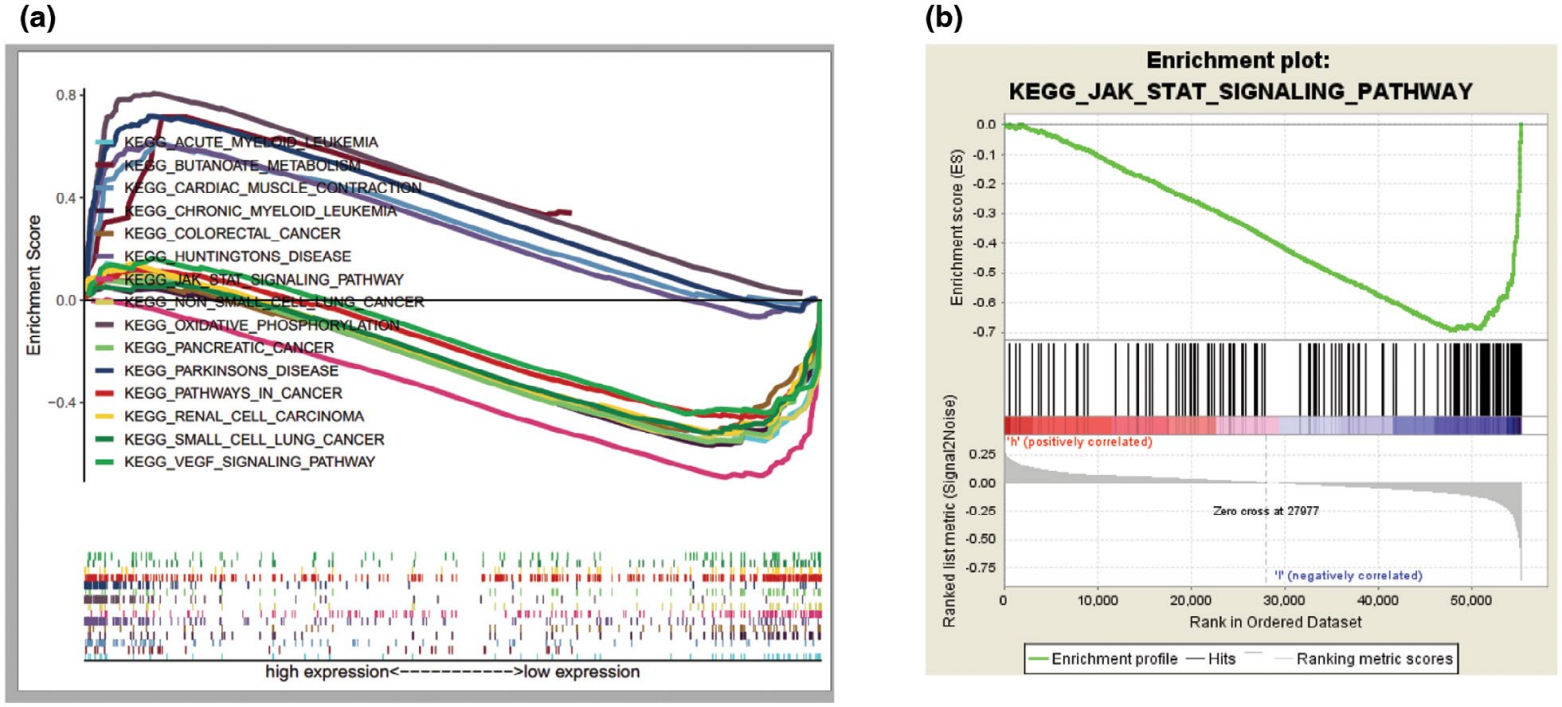
The enrichment plots from the GSEA results. (a) XCR1‐related signaling pathways from GSEA. (b) GSEA showed that the JAK/STAT signaling pathway is a regulatory target of XCR1. The GSEA enrichment plot identified values for normalized enrichment score (NES = −2.93 and nominal *P* value =0.)

GSEA analysis of low versus high expression of *XCR1* was used to explore the signaling pathways that are possibly associated with ccRCC. These results were presented in terms of the enrichment score (ES), NES, nominal (NOM) *P* value, and false discovery rate FDR q values (Figure 4a). Among the XCR1 high expression subgroup, only butanoate metabolism, cardiac muscle contraction, Huntington's disease, oxidative phosphorylation, and Parkinson's disease pathways were enriched, among which there is no classical tumor‐associated signaling pathway (Table 5). While the Janus kinase (JAK)/signal transducer and activation of transcription (STAT) pathway ranked first among all the downregulated pathways (Table 6) followed by natural killer cell‐mediated cytotoxicity, autoimmune thyroid disease, antigen processing and presentation, cell adhesion molecules (CAMs), et al, showing that XCR1 might be inversely and significantly correlated with JAK/STAT signaling (FDR <0.05, NOM *p* < 0.05; Figure 4b).

**Table 5:** Top five signaling pathways enriched in the *XCR1* upregulation group.

Rank	Name of pathway	ES	NES	NOM p‐val	FDR q‐val
1	HUNTINGTONS_DISEASE	0.616067	2.251294	0	0.01076
2	CARDIAC_MUSCLE_CONTRACTION	0.614087	2.145381	0	0.022747
3	OXIDATIVE_PHOSPHORYLATION	0.806736	2.122762	0	0.020029
4	BUTANOATE_METABOLISM	0.714311	2.092098	0.004141	0.019135
5	PARKINSONS_DISEASE	0.716557	2.081168	0.001957	0.017659

FDR, false discovery rate; NES, normalized enrichment score; NOM, nominal.

**Table 6:** Top 10 signaling pathways enriched in the *XCR1* downregulation group.

Rank	Name of pathway	Es	NES	NOM p‐val	FDR q‐val
1	JAK STAT SIGNALING PATHWAY	−0.6941993	−2.92982	0	0
2	NON SMALL CELL LUNG CANCER	−0.55117565	−1.92028	0.010204	0.022405
3	ACUTE MYELOID LEUKEMIA	−0.5652863	−1.91111	0.013834	0.023524
4	VEGF SIGNALING PATHWAY	−0.45163378	−1.90325	0	0.02442
5	CHRONIC MYELOID LEUKEMIA	−0.5741395	−1.9007	0.014553	0.024273
6	PANCREATIC CANCER	−0.56183416	−1.88966	0.02439	0.025081
7	PATHWAYS IN CANCER	−0.4609015	−1.88757	0.014315	0.024817
8	SMALL CELL LUNG CANCER	−0.5270194	−1.83966	0.022222	0.030835
9	COLORECTAL CANCER	−0.5336979	−1.78094	0.027083	0.043837
10	RENAL CELL CARCINOMA	−0.53083134	−1.76747	0.034836	0.045633

FDR, false discovery rate; NES, normalized enrichment score; NOM, nominal.

### 3.5 Knockdown of *XCR1* increases ccRCC cells proliferation and migration and inhibits apoptosis

**FIGURE 5:**
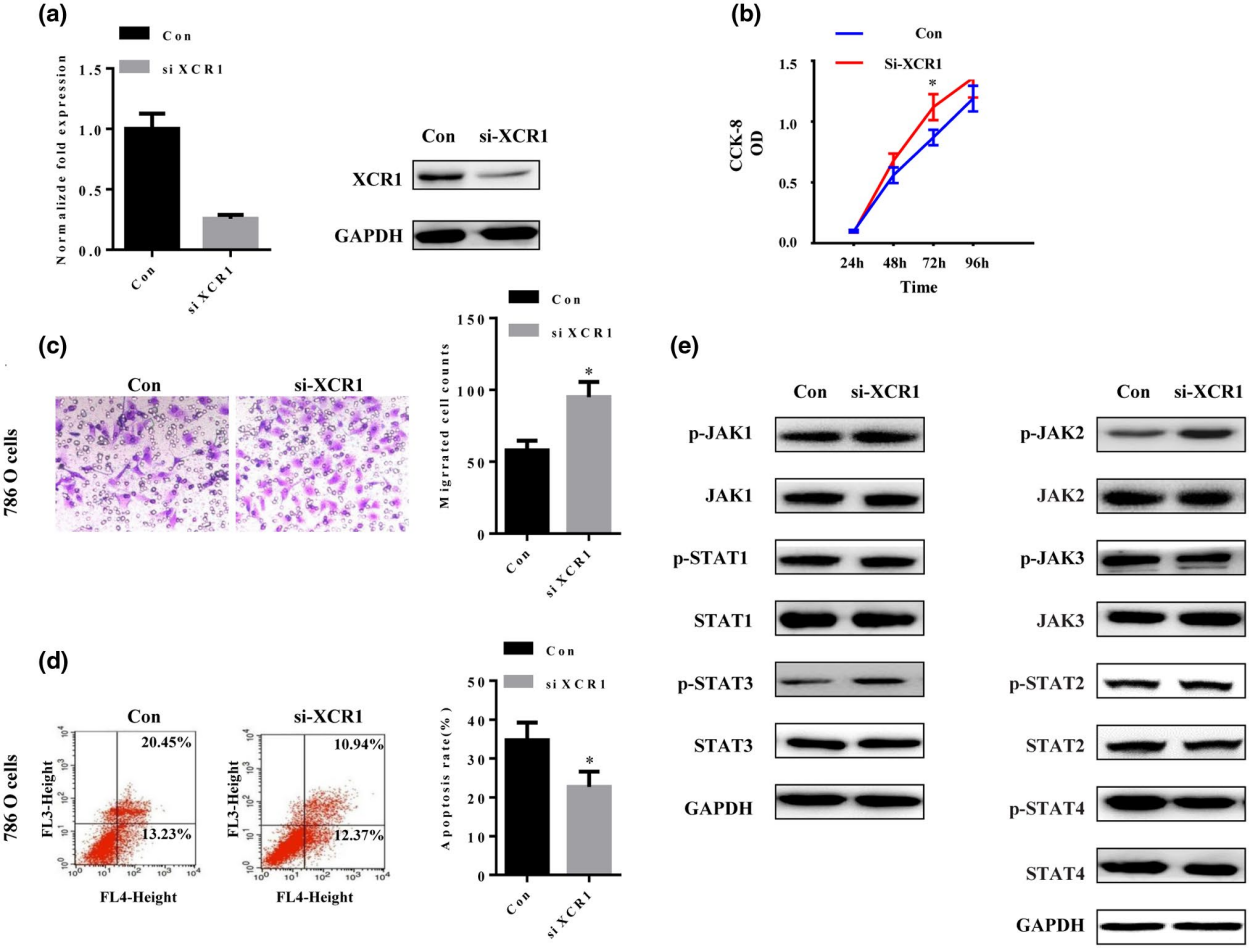
*XCR1* knockdown increases ccRCC cell proliferation and migration, inhibits the apoptosis via JAK2/STAT. (a) The expression of XCR1 in 786‐O cell lines transfected with siXCR1 was evaluated using qRT‐PCR and western blotting. Blots are representative of three experiments and were re‐probed for GAPDH to verify equal loading (b) Downregulation of *XCR1* caused a significant growth increase of 786‐O cells, as revealed by CCK‐8 assay. SiXCR1 versus control group, *p* < 0.05. Values shown are the mean absorbance ±SD for five wells from one experiment and are representative of three independent experiments. (c) Transwell migration assay using the siXCR1 vector. After the knockdown of *XCR1*, cell migration increased significantly. The migrated cells were stained with crystal violet and counted. Bar graph of the Transwell migration assay representing the mean value ±SD from three independent experiments. *p < 0.05. Scale bars: 50 μm. (d) siXCR1 and control 786‐O cells were stained with Annexin V‐APC and 7‐AAD for flow cytometry analysis. Shown are representative images of three independent experiments. Bar graph of the flow cytometry analysis showing the mean ±SD from three independent experiments. *p < 0.05. (e) Western blot analysis of key molecules of JAK/STAT pathways in 786‐O cells with or without XCR1 knockdown.

*XCR1* is abnormally highly expressed in ccRCC; therefore, we set to silence its expression in ccRCC cells by a siRNA. The indicated vectors were transfected into human 786‐O RCC cells as described previously (Zheng et al., 2016).Then the expression of XCR1 at both nucleic acid and protein levels was tested by qRT‐PCR and western blotting assays, respectively. It was shown XCR1 expression had been reduced by 75% compared with that in the control group (Figure 5a). Additionally, CCK8 and Transwell migration results showed that, after knockdown of *XCR1*, the RCC cells proliferation and migration were shown to be significantly increased (Figure 5b,c). Moreover, flow cytometry result indicated that the apoptosis rate of the siXCR1 group decreased significantly compared with those in the control group (Figure 5d, *p* = .026).

According to the GSEA enrichment result, XCR1 was significantly inversely correlated with JAK/STAT signaling; therefore, we explored the association between XCR1 and key molecules of JAK/STAT pathways in 786‐O cells. In the siXCR1 group, the levels of phosphorylated (p)‐JAK2 and p‐STAT3 were significantly upregulated which will then activate the JAK/STAT signaling pathway, while those of p‐JAK1, p‐JAK3, p‐STAT1, p‐STAT2, and p‐STAT4 were slightly changed or unchanged, indicating that JAK2 and STAT3 are the key proteins related to the indicated biological roles of XCR1 in ccRCC cells (Figure 5e).

## 4 DISCUSSION

RCC, which is the main type of kidney cancer, is the eighth leading cause of cancer‐related death (Balkwill, 2004). RCC occurs as multiple histologically and genetically heterogeneous subtypes, among which ccRCC represents 80% of RCC cases (Shang et al., 2007) and accounts for ~3% of adult cancer. The ccRCC tumor cells are highly metastatic and invasive, and can withstand radiation and chemotherapy. Thus, there is an urgent need to identify valuable diagnostic markers for RCC and to find potential targets that might lead to more effective molecular therapy for ccRCC.

In the present study, we found that XCR1 is overexpressed in ccRCC tumors, according to the analysis of data from the TCGA database. Cox regression and Kaplan–Meier analysis showed that patients with higher XCR1 expression were associated with better prognosis; therefore, XCR1 may be a potential prognostic biomarker for ccRCC. Moreover, these results were verified in clinical samples collected from patients who underwent radical or partial nephrectomy. Patients with higher XCR1 expression had longer overall survival, which prompted us to speculate it might be related to the role of the XCR1 axis in the immune system. It has been reported that a combination of cytokine and chemokine receptors could inhibit tumor formation and growth by stimulating or activating T lymphocyte cytotoxicity, natural killer (NK) cells, and dendritic cells. In addition, an immune vaccine modified with interleukin (IL2)‐XCL1 showed an obvious anti‐neuroblastoma effect (Shang et al., 2011). The combination of IL10 and XCL1 could stimulate or activate NK cells and cytotoxic T cells, which could promote the production of tumor necrosis factor alpha (TNFα) and inhibit the production of vascular endothelial growth factor (VEGF) and matrix metalloproteinase (MMP2), thereby achieving an anti‐cancer therapeutic effect. The XCL1/XCR1 axis plays an important role in the immune system, including the regulation of dendritic cell (DC)‐mediated cytotoxic immune response, establishment of thymus self‐tolerance, and the production of regulatory T cells (Siegel et al., 2018). However, the underlying molecular mechanism remains unclear, and the phenomenon is worth further investigation. He's research group identified prognostic microenvironment‐related genes in ccRCC and found that the ESTIMATE algorithm‐based stromal and immune scores might be a credible indicator of cancer prognosis (Subramanian et al., 2005). Moreover, they found that XCR1 alone, or with IL10, could be a potential key regulator of the tumor microenvironment (Subramanian et al., 2005), which could also represent corroborative evidence for our speculation concerning XCR1’s involvement in ccRCC. The exact role of XCR1 in ccRCC, especially its prognostic value, requires further study.

Recent studies have also demonstrated the importance of XCL1/XCR1 in cancer cell proliferation, migration, and invasion. XCR1 could stimulate migration, invasion, and proliferation via the extracellular signal‐related kinase (ERK)1/2 signaling pathway in oral epithelial cells (Jemal et al., 2010). The expression of XCR1 is inhibited in human breast cancer cells in vitro and in vivo through inhibiting the mitogen‐activated protein kinase (MAPK) and phosphatidylinositol‐4,5‐bisphosphate 3‐kinase (PI3K)/AKT/mammalian target of rapamycin (mTOR) signaling pathway (Wang et al., 2015). In the present study, GSEA demonstrated that the JAK/STAT signaling pathway was the most downregulated pathway related to XCR1 in ccRCC. Regulation of the JAK/STAT signaling pathway during tumorigenesis might have dual functions. On the one hand, the blockade of STAT interrupts the transmission of signals arriving at the cell nucleus, which would cause genetic disorders and tumorigenesis. O n the other hand, large amounts of target genes are transcribed as a result of abnormal STAT activation, which could also cause tumorigenesis (Yamazaki et al., 2010; Yang et al., 2017). It is reported that the abnormal activation of STAT3 promotes in tumor formation, while STAT1’s abnormal activation in tumors triggers the body's anti‐tumor mechanism (Yanru et al., 2018). Restoring the expression of JAK or STAT1 might strikingly increase the susceptibility of RCC to interferon‐alpha (IFN‐α) and could be a new strategy to improve the response of RCC to IFN‐α treatment (Zhang et al., 2001). The JAK/STAT pathway should, therefore, be an appropriate target for the treatment of RCC (Zheng et al., 2016), which may be involved in the regulation of XCR1 on ccRCC.

We then used the 786‐O cell line to detect the potential role of XCR1 on ccRCC cell proliferation, migration, and apoptosis. After knockdown of *XCR1*, cell proliferation and migration increased notably, while the apoptosis rate decreased significantly. As a result, our study demonstrated that XCR1, a potential prognostic biomarker for ccRCC, could decrease tumor cell proliferation and migration, and increase apoptosis.

## 5 CONCLUSION

Based on the data analysis of the TCGA data and clinical samples collected from Xinqiao Hospital, we found that XCR1 was overexpressed in ccRCC, and its higher expression was associated with longer overall survival. Downregulation of XCR1 promoted ccRCC cell proliferation and migration, and might decrease RCC cell apoptosis. Our data suggested that XCR1 could be used as a potential biomarker with good prognostic value for clinical application in ccRCC in the future.

## CONFLICT OF INTEREST

The authors report no conflicts of interest in this work.

## AUTHOR CONTRIBUTIONS

QNG and YFL conceived and designed the study. CX and CY analyzed the data and prepared the figures and manuscript. WLF, XFT, and WYL performed the immunohistochemistry assay. All authors have read and approved the final version of the manuscript.

## Data Availability

The data of this study are available from the corresponding author upon reasonable request.
